# Using transformer-based models for Vietnamese language detection

**DOI:** 10.1371/journal.pone.0342898

**Published:** 2026-02-13

**Authors:** Son Tran, Phuoc Tran

**Affiliations:** Natural Language Processing and Knowledge Discovery Research Group, Faculty of Information Technology, Ton Duc Thang University, Ho Chi Minh City, Vietnam; University of Jaen: Universidad de Jaen, SPAIN

## Abstract

This paper introduces a solution to the problem of detecting whether a sequence of text is Vietnamese based on its orthography and contextual features. For those unfamiliar with the language, it is known that understanding the meaning of certain texts can be challenging, since Vietnamese is a complex language that uses Latin characters with diacritics, and many of its words rely heavily on accent marks for semantic distinction. In this paper, we provide insight into how these characteristics influence Transformer-based natural language processing models and propose an approach to address this issue. Transformer-based models are selected due to their superior performance compared to earlier architectures such as RNNs and LSTMs, as well as their widespread application in state-of-the-art NLP systems (GPT, BERT, T5). We examine the specific challenges posed by Vietnamese orthography and word formation, and propose a solution that enhances the model’s ability to distinguish Vietnamese text. Our approach is evaluated on a benchmark dataset, demonstrating high accuracy and robustness in Vietnamese text detection, outperforming conventional methods. The results confirm that Transformer-based models can effectively learn orthographic and contextual patterns in Vietnamese, contributing to improved language identification and multilingual NLP processing.

## 1. Introduction

### 1.1. Motivation

Among the languages employing the Latin alphabet, Vietnamese stands out due to its tonal system and extensive use of diacritics. These features are fundamental to the language as they not only influence pronunciation but also differentiate the meanings of words, making them essential in both spoken communication and computational processing.

The linguistic complexity of Vietnamese poses challenges for Natural Language Processing (NLP). Processing text without diacritics can lead to misinterpretation, as homographs become indistinguishable without context. Furthermore, the isolating nature of Vietnamese, where meaning is primarily derived from word order and context rather than inflection, adds another layer of complexity. Interpreting, understanding, and detecting both non-diacritical and diacritical Vietnamese can be challenging, often leading to confusion for readers or recipients.

Transformer-based models such as XLM-RoBERTa [1], E5 [2], and the Vietnamese-specific BARTPho [3] offer promising solutions by excelling at capturing contextual dependencies within text. These models have also been successful in multilingual tasks, including applications in Vietnamese language processing. However, existing studies primarily focus on diacritical Vietnamese text or formal language, leaving a significant gap in addressing the non-diacritical text prevalent in informal contexts. This gap between the language’s linguistic complexity and the capabilities of current NLP tools restricts the accessibility and adaptability of Vietnamese in the digital ecosystem.

This gap becomes more evident when examining the limitations of current approaches. Many traditional methods rely on rule-based diacritic restoration or statistical models trained exclusively on diacritic-rich corpora, which fail to generalize when diacritics are absent. Even Transformer-based models, though powerful, are often fine-tuned on clean, formal Vietnamese text. As a result, they struggle to disambiguate homographs in diacritic-less settings, where multiple valid interpretations exist. Moreover, non-diacritical text frequently appears in informal communication such as SMS, social media posts, or search queries, which differ significantly from the training data of most existing models. These mismatches lead to performance drops, semantic ambiguity, and reduced usability of Vietnamese NLP tools in real-world applications.

In this context, the motivation for this paper is clear: there is a critical need to bridge the gap between the linguistic intricacies of Vietnamese and the limitations of existing computational models. This paper aims to prioritize the analysis and processing of non-diacritical Vietnamese text, as we seek to overcome existing shortcomings in Vietnamese NLP, ensuring that the language remains accessible, adaptable, and efficiently processed in modern computational applications. By doing so, the paper contributes to the development of robust Vietnamese NLP tools that can better serve the needs of both researchers and end users in a digital-first world.

### 1.2. Objective

This paper seeks to extend the boundaries of knowledge and advance the computational processing of the Vietnamese language through the application of state-of-the-art Transformer-based models such as XLM-RoBERTa-Base, E5, and BARTPho. This paper addresses two primary problems:

- The first concerns the identification and lemmatization of Vietnamese texts that have been accurately spell-checked.
- The second involves the identification and simultaneous lemmatization of diacritic-less Vietnamese text.

These challenges are pertinent to core issues in Vietnamese NLP due to the characteristics of the language, including its tonal system and highly productive word-compounding structures. To achieve these goals, the research intends either to use existing benchmark datasets or construct new, task-specific datasets that more accurately capture the linguistic nuances of Vietnamese. Transformer-based models will be implemented on these datasets to systematically evaluate their effectiveness in addressing the aforementioned problems. Following the performance evaluation, we will be able to offer a detailed interpretation of each model’s strengths and limitations, thereby contributing to a deeper understanding of their behavior across various Vietnamese NLP tasks.

In addition, the paper aims to develop a practical application that integrates the findings from this study. This application will serve as a proof-of-concept system demonstrating the capability of Transformer-based models to process Vietnamese text in both diacritic and non-diacritic forms, offering a valuable tool for tasks that require advanced language understanding and processing. By accomplishing these goals, this paper hopes to make a significant contribution to the field of Vietnamese NLP, addressing persistent challenges and establishing a solid foundation for future research initiatives.

The remainder of this paper is structured as follows: Section II reviews related work and fundamental concepts, including recent studies on Vietnamese natural language processing (NLP) and Transformer-based approaches such as XLM-RoBERTa, BARTPho, and E5. Section III describes the methodology, detailing the overall framework, dataset preparation, and model implementation. Section IV presents the experimental setup, dataset descriptions, evaluation metrics, and results analysis, providing insights into model performance through tables and figures. Finally, Section V introduces a demonstrative application that showcases the practical use of Transformer-based models for Vietnamese text processing in real-world scenarios.

## 2. Related work

Recent advancements in natural language processing (NLP) have paved the way for developing Vietnamese-specific models and tools. This section features prominent studies that form the basis of this research, highlighting their contributions and distinguishing their focus from our paper’s objectives.

Recent research on Transformer-based models has significantly advanced natural language processing (NLP) for the Vietnamese language. One key area of focus is the integration of Transformer-based Contextualized Language Models (CLMs) with neural networks for tasks like Natural Language Inference (NLI). This integrated approach has demonstrated notable progress by delivering strong performance while maintaining computational efficiency, making it suitable for real-world applications where resource constraints are important. The combination of CLMs and neural networks enhances contextual representation learning, an area often underexplored in previous studies [1].

Another significant contribution comes from the integration of BERT with Transformer models for Vietnamese spelling correction. This method capitalizes on the power of contextual embeddings from BERT [4], combined with the sequence-to-sequence capabilities of Transformer architectures. It effectively handles common Vietnamese spelling issues—including those caused by dialectal variation and informal abbreviations—while achieving state-of-the-art results for spelling correction tasks [5]. This direction of research explicitly addresses challenges unique to Vietnamese orthography, unlike approaches developed for languages with simpler writing systems.

In the domain of hate speech detection, the ViHateT5 model has emerged as a notable contribution. It employs a text-to-text Transformer architecture, specifically trained on the VOZ-HSD dataset, which is designed to tackle the complexities of hate speech in Vietnamese [6]. By incorporating domain-specific pre-training, the model demonstrates substantial improvements across multiple benchmarks, highlighting the importance of specialized training data in enhancing the performance of NLP models in specific contexts. This work further reinforces the capability of Transformer models to address sensitive and context-dependent Vietnamese language tasks.

Furthermore, the ViT5 model, a Transformer-based text-to-text architecture, has shown remarkable performance in Vietnamese text generation tasks. Evaluated on downstream applications such as abstractive text summarization and Named Entity Recognition (NER), ViT5 outperforms existing models, achieving new state-of-the-art results for Vietnamese text summarization tasks. These results illustrate the adaptability and effectiveness of the text-to-text paradigm, particularly for languages with complex syntactic structures like Vietnamese [7].

In addition to these task-specific applications, several studies have focused on improving pre-trained language models for Vietnamese NLP. For example, ViDeBERTa [8], based on the DeBERTaV3 architecture, incorporates advanced techniques like Gradient-Disentangled Embedding Sharing (GDES) [9], improving the model’s performance in tasks such as Part-of-Speech tagging and Named Entity Recognition. By integrating additional self-supervised learning tasks, ViDeBERTa establishes new benchmark performance across multiple Vietnamese NLP tasks. Similarly, ViSoBERT, a pre-trained model developed for Vietnamese social media text processing, excels in handling informal language, emojis, and teencode, which are common in platforms like Facebook and TikTok. Its customized tokenizer enables more accurate interpretation of informal Vietnamese, ensuring better contextual understanding and more efficient processing of Vietnamese social media content [10].

While these advancements have made significant strides in improving Vietnamese NLP, challenges remain, particularly in handling non-diacritical Vietnamese text. PhoNLP, a multi-task learning model based on BERT, provides a comprehensive toolkit for dependency parsing, Part-of-Speech tagging, and NER. However, it does not specifically address the processing of non-diacritical Vietnamese text, leaving an important practical gap unaddressed for informal communication [11]. This issue is also highlighted in the work on XLM-RoBERTa, a multilingual Transformer model trained on a vast dataset spanning multiple languages. While XLM-RoBERTa has proven effective for cross-lingual tasks, including classification and token-level tasks, its performance on Vietnamese remains limited by challenges related to tonal encoding and orthographic variation [1].

Furthermore, models like VietOCR have combined Convolutional Neural Networks (CNNs) with Transformer architectures to tackle Optical Character Recognition (OCR) for Vietnamese [12]. Trained on a large dataset containing handwritten text and scanned documents, VietOCR achieves high accuracy in recognizing Vietnamese text, demonstrating the power of combining visual and textual processing capabilities in NLP tasks [13]. Similarly, ViLBERT, a multi-modal model, has been pre-trained on a large dataset of images and captions, enabling it to process both visual and textual inputs. This reflects a broader trend toward integrating multimodal learning frameworks to enhance Vietnamese language understanding [14].

The reviewed literature thus confirms the power of Transformer-based architectures for Vietnamese, yet also reveals a shared limitation: a predominant focus on formal, diacritic-rich text, leaving the challenge of identifying informal, non-diacritical Vietnamese largely unaddressed. The following sections detail our methodology for tackling this specific problem, beginning with the assembly of a suitable dataset.

More recent efforts, including VinaLLaMA and PhoGPT-4B-Chat, illustrate the growing trend of large-scale Vietnamese generative models that rival multilingual LLMs in question answering and reasoning tasks [15,16]. Likewise, LaVy introduces a multimodal framework for Vietnamese [17]. These models highlight the increasing sophistication of Vietnamese NLP research; however, they are largely designed for formal or diacritic-rich inputs and benchmarked on curated datasets, rather than the noisy, diacritic-less text prevalent in everyday communication.

This presents a critical challenge: Vietnamese NLP models often fail to generalize or accurately process text devoid of tone marks. To bridge this gap, our paper also focuses on developing a Transformer-based model capable of effectively handling non-diacritical Vietnamese text. Furthermore, we aim to establish a more comprehensive dataset of non-diacritical Vietnamese text, which is currently underrepresented in existing benchmarks. Our goal is to enhance the robustness and practical applicability of Transformer-based Vietnamese NLP across both formal and informal communication settings.

## 3. Solution

### 3.1. Overview

**Fig 1:**
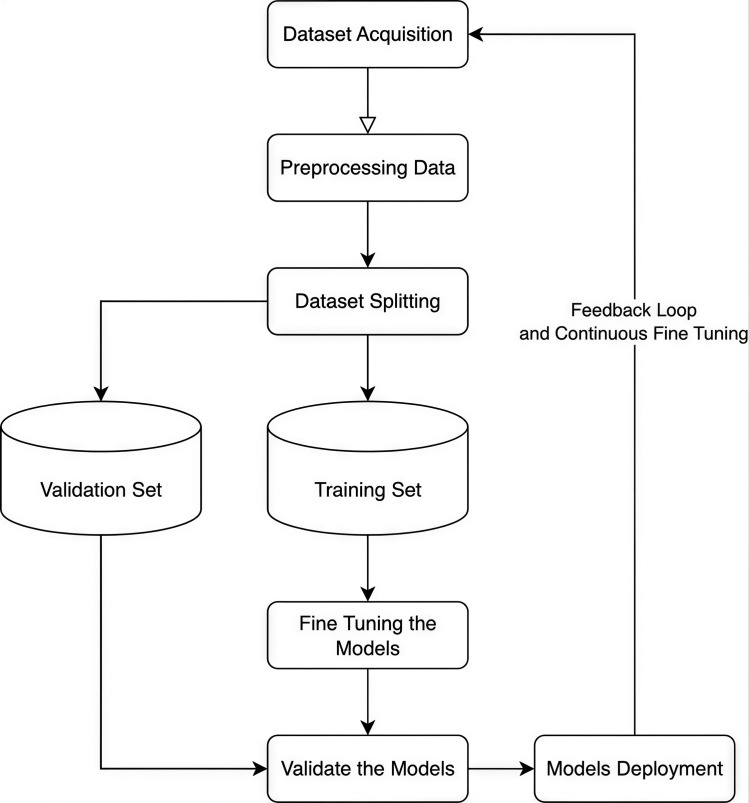
Comprehensive workflow of the proposed solution, illustrating the iterative pipeline from dataset acquisition and preprocessing to model fine-tuning, validation, and the continuous feedback loop for deployment.

The overall architectural framework is depicted in Fig 1, which illustrates the end-to-end lifecycle of the solution. This iterative pipeline integrates dataset acquisition, rigorous preprocessing, and model fine-tuning to ensure the system evolves through a continuous feedback loop. Starting with dataset acquisition and cleaning to ensure high-quality input. The data is split into training, validation, and test sets before applying it to a pretrained model. After preparing the data with tokenization and attention masks, the model is fine-tuned through a structured training pipeline. Validation and testing are conducted to optimize the best performance, followed by deployment for real-world applications.

### 3.2. Solution

#### 3.2.1. Data acquisition.

Data is acquired from both publicly available datasets and self-collecting datasets. Publicly available datasets used in this research include Binhvq News Corpus [18], vi-error-correction-2.0 [19] and OPUS Tatoeba [20]. The collection and analysis of these open-source datasets were performed in strict accordance with their respective distribution licenses. Details of each dataset are given in Section 4.

Datasets we use in this solution will include publicly available datasets combined with text crawled mostly from news listing websites such as VNExpress, since for both XLM-RoBERTa and E5, labelled datasets are preferred for optimal performance. After datasets are sufficiently collected, we will then divide the dataset into two positive and negative sets. The positive set contains Vietnamese sample texts, while the other contains texts such as English, French, Spanish, and other non-Vietnamese languages. In the positive set, a percentage of sample texts will have their diacritics removed to ensure the models’ robustness against unaccented Vietnamese text.

a) Binhvq News Corpus:

This dataset is available on GitHub, created by Vuong Quoc Binh [18]. According to the author, the full dataset is extracted from 14,896,998 articles on the Internet, from which the titles contain 10,787,976 sentences. Including the descriptions and bodies, the dataset comprises 111,274,300 sentences in total, amounting to more than 18.6 GB of storage. Table 1 shows the structure of the dataset.

**Table 1:** Structure of raw Binhvq News Corpus.

Index	Text	Meaning
0	Chây ì nộp phạt nguội.	Delaying on payment of traffic fines
1	Cháu đòi tiền cơm, dì đòi tiền nhà.	Siblings ask for meal money, aunts ask for rent
2	Đà Nẵng nghiên cứu tiện ích nhắn tin khi vi phạm đến chủ phương tiện.	Da Nang is researching a feature to notify vehicle owners via text message when a violation occurs.
3	Khó xử vụ mẹ 70 tuổi trộm xe hơi của con gái.	Awkward situation: 70-year-old mother steals her daughter’s car.
4	Thay đổi về đăng ký, chuyển nhượng xe từ 12/2 bạn cần biết.	Changes in vehicle registration and ownership transfer from February 12 that you need to know.

The sentences in the dataset are processed through a basic preprocessing procedure, including sentence segmentation with PunktSentenceTokenizer, restoring corrupted characters during HTML-to-text conversion, removing duplicate or near-duplicate sentences, and normalizing Unicode characters using the Normalization Form Canonical Composition (NFC) algorithm, where characters are decomposed and then recomposed according to canonical equivalence. We use this dataset to insert records containing Vietnamese words into our own dataset.

b) Vi-error-correction-2.0:

This dataset is available on Hugging Face, created by bmd1905 [19]. The full dataset consists of 2,728,488 records, with two columns: input and output. The input column contains Vietnamese texts with misspellings, while the output column provides their corrected forms. We use this dataset to insert records that contain misspelled Vietnamese words. The structure of this dataset is shown at Table 2.

**Table 2:** Structure of raw vi-error-correction-2.0 dataset.

Index	Text	Output	Meaning
0	Bắn ằng súNg gì?	Bắn bằng súng gì?	Which gun is used?
1	Đặc điểm nơi gây án?	Đặc điểm nơi gây án?	Which gun is used?
2	ĐẶc điểm nơi gâUy án?	Đặc điểm nơi gây án?	Which gun is used?
3	Đặc diKm nơi gây án?	Đặc điểm nơi gây án?	Which gun is used?
4	Đặc điểm oơi gây áN?	Đặc điểm nơi gây án?	Which gun is used?

c) OPUS Tatoeba:

This dataset is available on Hugging Face, created by Team wecover [20]. The full dataset consists of 84 subsets, each labeled with a corresponding country code. Combined together, they form a dataset containing 6,379,706 records, with a total size of 417 MB. We use this dataset to insert records that are not Vietnamese words into our dataset. Each subset includes a sentence1 field written in lang1, along with its translation into lang2 in the sentence2 field. Details of this dataset is shown at Table 3.

**Table 3:** Structure of raw OPUS Tatoeba dataset.

	Sentence1	Sentence2	Guid	Lang1	Lang2	Actual_meaning
0	Ek het ‘n bierbuik.	عندي بطن الجعة.	0	Afrikaans	Arabic	I have a beer belly.
1	Ek wil ‘n rooi kar hê.	Vull un cotxe vermell.	2	Afrikaans	Catalan	I want a red car.
2	Hy is ‘n ou man.	Ell és un home vell.	0	Afrikaans	Catalan	He is an old man.
3	Hierdie tafel is van hout gemaak.	Tento stůl je ze dřeva.	1	Afrikaans	Czech	This table is made of wood.
4	Wat moet ek hom vir Valentynsdag gee?	Was sollte ich ihm zum Valentinstag schenken?	1317	Afrikaans	German	What should I give him for Valentine’s Day?

We use this dataset to improve our model’s robustness in detecting text that is not Vietnamese, especially in cases where other languages also contain accented characters, such as Spanish, Slavic languages, or Catalan.

With the three given datasets, we can combine them to create a labelled dataset containing both Vietnamese and non-Vietnamese texts. For Vietnamese texts, we apply a Unicode-diacritic removal function to generate non-accented variants, allowing the model to better handle unaccented Vietnamese inputs. We also include misspelled samples from the vi-error-correction-2.0 dataset to further enhance robustness.

**Fig 2:**
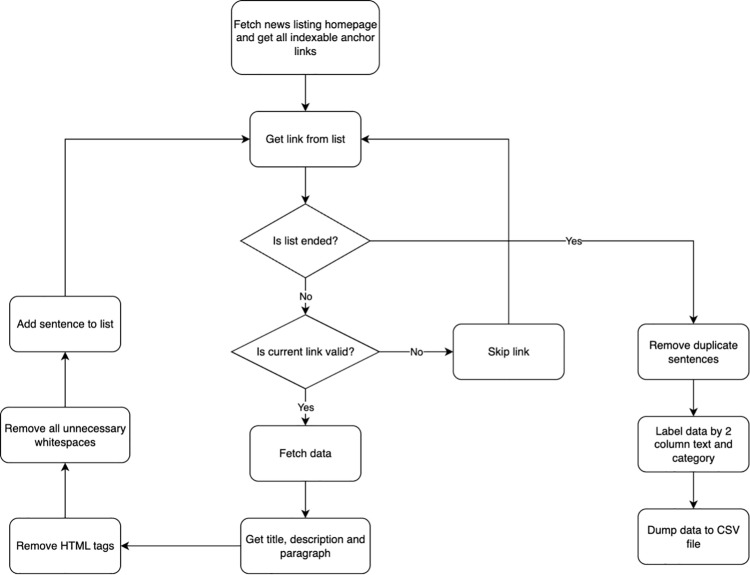
Data acquisition flowchart detailing the automated crawling mechanism, including link validation, content extraction, HTML cleaning, and deduplication logic for constructing the raw corpus.

d) Self-collecting datasets:

Self-collecting datasets are composed of information crawled from news listing websites. Using Python, we developed a program to run as a scheduled CRON job every Monday, Wednesday, and Friday to crawl these websites. This periodic scheduling ensures that the data remains up to date while minimizing manual intervention. The crawler currently supports VNExpress in both its Vietnamese and English versions. We confirm that the data collection and analysis methods complied with the terms and conditions of the source website. Specifically, the crawler adhered to the robots.txt protocols of VNExpress, extracting only publicly accessible information for academic research purposes without bypassing any access controls. The automated mechanism for traversing news repositories and extracting textual content is visually detailed in Fig 2. As shown in the diagram, the crawler employs a systematic logic to validate anchor links, strip HTML artifacts, and perform strict deduplication before labeling the data for the training corpus.

The process of gathering and organizing textual data can be divided into a structured pipeline that combines web scraping, data validation, cleaning, and removing redundant data. The purpose of this operation is to extract relevant information from the homepage and its subsequent links, clean it to a standardized format, and organize the content for further analysis.

It begins with fetching anchor links from the VnExpress homepage. At this stage, the crawler identifies all indexable links using predefined HTML class selectors, such as section_topstory and section_container. These classes typically contain article summaries or highlights. To parse these elements, the crawler utilizes Selenium, a web scraping automation tool, to interact with dynamic web pages, and BeautifulSoup, a library for parsing HTML content. All extracted URLs are collected into a list, where duplicates are removed, ensuring only unique links proceed to the next stage.

Once the links are fetched and duplicate links are removed, the crawler validates each one to determine whether it meets the required criteria for further processing. Invalid or broken links are skipped, while valid ones undergo a detailed scraping process. In this step, the script extracts the core components of each article—title, description, and content paragraphs—by targeting key HTML structures rather than relying solely on broad element selection. This selective extraction ensures that only meaningful and relevant data is collected.

Following data extraction, the pipeline emphasizes data cleaning. During this stage, unnecessary HTML tags and redundant whitespaces are removed. Sentences are further standardized to ensure consistency in formatting. Additionally, the pipeline performs rigorous deduplication, removing repeated sentences both within individual articles and across the dataset to maintain data integrity. The cleaned data is then labeled into a structured format, typically organized into two columns: one for the textual content and the other for the associated category (language). This standardized labeling approach enhances compatibility with downstream processing tasks, such as machine learning or natural language processing (NLP) applications.

#### 3.2.2. Labeling and grouping datasets.

After collecting data from the datasets mentioned above, we proceed to clean the data and turn them into the needed form to prepare for model training. While doing this, we combine both grouping the datasets and cleaning the data. The cleaning process consists of removing noisy, duplicated data and normalizing. Data between datasets are formatted differently, so it is necessary to format them into one format, then proceed to group them into one big dataset. A detailed description of the structure of each dataset is provided in Section 4. After unifying their formats, all datasets are transformed into a standardized schema consisting of two columns:

- Text: Contains the text sample.
- Category: Contains the category of text, including “Vietnamese”, “Not Vietnamese”, “Potential Vietnamese”. The label “Potential Vietnamese” is assigned to samples in which Vietnamese diacritics have been removed or the text contains misspellings.

Structure transformation of each dataset is shown as below:

- Binhvq News Corpus: This dataset consists only of texts in Vietnamese, so all records are labeled as “Vietnamese” in the category column.
- *vi-error-correction-2.0:* This dataset contains misspelled Vietnamese text. Data is formatted as tabular with two columns, input (misspelled text) and output (corrected text). However, since each output text may correspond to multiple input texts, it is necessary to remove duplicate records to avoid bias. We only retain the input texts and label them as “Potential Vietnamese.”
- *OPUS Tatoeba:* This dataset contains mostly non-Vietnamese texts. The table consists of two columns, sentence1 and sentence2, each written in its corresponding lang1 and lang2 language. With this structure, we can extract both sentences as separate non-Vietnamese samples, effectively doubling the amount of usable data.
- *Crawled dataset:* This dataset contains two language versions: Vietnamese and English. Columns of the dataset consist of text and lang, allowing us to categorize each text based on its corresponding language, with its structure is shown at Table 4 and Table 5.

**Table 4:** Structure of raw vietnamese crawling dataset.

Index	Text	Lang	Meaning
0	Quá trình sắp xếp kiện toàn bộ máy, phải đảm...	vi	The process of reorganizing and consolidating the apparatus must ensure...
1	Mức thưởng Tết Dương lịch ở Hà Giang cao nhất...	vi	The highest New Year bonus in Ha Giang is...
2	Kỳ thi đánh giá của Bộ Công an	vi	The Ministry of Public Security’s assessment exam.
3	Đồng thời, nhu cầu tinh gọn bộ máy cũng phản á...	vi	At the same time, the need to streamline the apparatus also reflects...
4	Đấu giá 4,4 ha đất gần hồ Linh Đàm khởi điểm 8...	vi	Auction of 4.4 hectares of land near Linh Dam Lake starting at 8...

**Table 5:** Structure of raw english crawling dataset.

Index	Text	Lang
0	Choosing a reliable carpet contractor is key t...	en
1	Thus if a couple borrow 70% of the property’s...	en
2	The couple reportedly broke up in 2021, and Gu...	en
3	Addressing rumors that he was the third party...	en
4	Hong Kong actor Nicholas Tse and girlfriend si...	en

After converting all dataset structures into a unified format, we then proceed to merge them into a single dataset.

#### 3.2.3. Dataset acquisition.

The data acquisition phase involved ingesting the primary corpus from a structured CSV repository. The dataset comprises two principal features: “text” (the linguistic input) and “category” (the target label). Prior to processing, a rigorous inspection of the dataset’s dimensionality and structure was conducted to ensure alignment with the study’s machine learning objectives. Particular emphasis was placed on evaluating the overall quality, balance, and linguistic diversity of the corpus, as these factors are critical for minimizing systemic bias and ensuring the model’s ability to generalize to unseen data.

#### 3.2.4. Data preprocessing pipeline.

To transform the raw corpus into a standardized format optimized for Transformer-based architectures, a comprehensive preprocessing pipeline was implemented. This process addressed data quality issues through the following specific procedures:

- Whitespace Normalization: Regular expressions were employed to identify and eliminate redundant whitespace, ensuring consistency during the tokenization phase.
- Data Integrity Verification: Records containing missing or null values in the “text” field were excised to prevent propagation errors during training.
- Noise Reduction: To reduce dataset noise, entries containing fewer than five characters were filtered out, as such brief segments typically lack sufficient semantic context for effective classification.
- De-duplication: Duplicate records were removed to prevent the model from overfitting to frequent samples and to ensure the validity of performance metrics.
- Linguistic Standardization: A critical step for the Vietnamese subset involved text normalization using the underthesea toolkit [21]. This process ensured linguistic consistency by standardizing diacritics and accent placements.
- Character Filtering: The text was sanitized by removing non-standard characters, retaining only valid Vietnamese alphabetic characters, standard punctuation, and essential symbols.

This rigorous preprocessing regimen resulted in a refined, consistent dataset, thereby enhancing data reliability and strengthening the foundation for subsequent model training and evaluation phases. The final structure of the processed Binhvu News Corpus is presented in Table 6. Similarly, the standardized formats for the vi-error-correction-2.0, OPUS Tatoeba, and the Vietnamese crawled datasets are detailed in Table 7, Table 8, Table 9, and Table 10 respectively.

**Table 6:** Structure of Binhvq News Corpus after processed.

Text	Category	Meaning
Chây ì nộp phạt nguội.	vietnamese	Delaying on payment of traffic fines
Cháu đòi tiền cơm, dì đòi tiền nhà.	vietnamese	Siblings ask for meal money, aunts ask for rent
Đà Nẵng nghiên cứu tiện ích nhắn tin khi vi phạm đến chủ phương tiện.	vietnamese	Da Nang is researching a feature to notify vehicle owners via text message when a violation occurs.
Khó xử vụ mẹ 70 tuổi trộm xe hơi của con gái.	vietnamese	Awkward situation: 70-year-old mother steals her daughter’s car.
Thay đổi về đăng ký, chuyển nhượng xe từ 12/2 bạn cần biết.	vietnamese	Changes in vehicle registration and ownership transfer from February 12 that you need to know.

**Table 7:** Structure of vi-error-correction-2.0 after processed.

Text	Actual_meaning	Category
Bắn ằng súNg gì?	What kind of gun was fired?	potential vietnamese
Đặc đim Nơi gây án?	Characteristics of the crime scene?	potential vietnamese
Khi giáp mặ, uy hiếp, Thanh Nga và chồng bà ph...	When confronted and threatened, Thanh Nga and her husband...	potential vietnamese
Mkột loạt câu hỏi nghiệp vụ đặt ra.	A series of professional (investigative) questions were raised.	potential vietnamese
VàU những câu trả lời của nhóm liên bang đều k...	And the answers from the federal group were all...	potential vietnamese

**Table 8:** Structure of OPUS Tatoeba dataset after processed.

Text	Actual_meaning	Category
Ek het ‘n bierbuik.	I have a beer belly.	not vietnamese
Ek wil ‘n rooi kar hê.	I want a red car.	not vietnamese
Hy is ‘n ou man.	He is an old man.	not vietnamese
Hierdie tafel is van hout gemaak.	This table is made of wood.	not vietnamese
Wat moet ek hom vir Valentynsdag gee?	What should I give him for Valentine’s Day?	not vietnamese

**Table 9:** Structure of Vietnamese crawled dataset after processed.

Text	Meaning	Category
Quá trình sắp xếp kiện toàn bộ máy, phải đảm...	The process of reorganizing and streamlining the apparatus must ensure...	vietnamese
Mức thưởng Tết Dương lịch ở Hà Giang cao nhất...	The highest New Year bonus in Ha Giang is...	vietnamese
Kỳ thi đánh giá của Bộ Công an	The Ministry of Public Security’s assessment exam.	vietnamese
Đồng thời, nhu cầu tinh gọn bộ máy cũng phản á...	At the same time, the need to streamline the apparatus also reflects...	vietnamese
Đấu giá 4,4 ha đất gần hồ Linh Đàm khởi điểm 8...	Auction of 4.4 hectares of land near Linh Dam Lake starting at 8...	vietnamese

**Table 10:** Structure of English crawled dataset after processed.

Text	Category
Choosing a reliable carpet contractor is key t...	not vietnamese
Thus, if a couple borrow 70% of the property’s...	not vietnamese
The couple reportedly broke up in 2021, and Gu...	not vietnamese
Addressing rumors that he was the third party...	not vietnamese
Hong Kong actor Nicholas Tse and girlfriend si...	not vietnamese

#### 3.2.5. Dataset splitting.

The dataset is divided into two subsets:

- Training Set: Used to train the model and adjust its weights.
- Test Set: Used to evaluate hyperparameters and monitor the model’s performance during training.

This splitting process ensures that the model’s performance is measured objectively and that it does not simply memorize the training data. Although explicit splitting into train, validation, and test sets isn’t shown in the code, the slicing could represent selecting a subset for training or testing purposes. A more robust and standardized splitting procedure can be implemented later using utilities such as scikit-learn’s train_test_split to ensure proper dataset partitioning.

#### 3.2.6. Load pretrained transformer model.

Our diagram mentions loading pretrained Transformer models such as BARTPho, XLM-RoBERTa, or E5. While this is not explicitly part of our provided code, it would typically involve using libraries like Hugging Face Transformers. In practice, this stage includes initializing both the tokenizer and the pretrained model, ensuring compatibility with the input text format and downstream task. This step loads a pretrained model and tokenizer to prepare the text data for the Transformer-based model. Pretrained models reduce the computational cost and time required for training while providing state-of-the-art performance for many tasks. They allow transfer learning, where the knowledge from the pretrained model is adapted to a specific task using a smaller dataset.

#### 3.2.7. Prepare data for training.

The raw input data is then converted into a format suitable for the transformer model:

- Tokenization: Breaking down the text into tokens that the model can understand.
- Attention Masks: Specifying which tokens should be attended to and which should be ignored (e.g., padding tokens).
- Input IDs: Converting tokens into numerical representations based on the model’s vocabulary.

This step ensures that the input data aligns perfectly with the requirements of the transformer model, allowing it to process and learn effectively.

#### 3.2.8. Model training.

This step involves configuring the parameters and settings for the training process, including:

- Hyperparameters: Key parameters like learning rate, batch size, and number of epochs are defined to control how the model learns.
- Optimizer: Algorithms like Adam or SGD are selected to minimize the loss function and adjust model weights efficiently.
- Learning Rate Scheduler: A strategy to adjust the learning rate dynamically during training, ensuring faster convergence and better results.

A well-configured pipeline is essential to achieve high performance without overfitting or underfitting.

#### 3.2.9. Model Training.

During training, the model iteratively learns patterns in the data by minimizing a predefined loss function. This involves multiple forward and backward passes, where:

- In the forward pass, predictions are made based on the input data.
- In the backward pass, gradients are calculated, and weights are updated to minimize errors.

Training often requires significant computational resources, and regular checkpoints are saved to ensure progress is not lost.

#### 3.2.10. Validation Loop.

As the model trains, its performance is evaluated on the validation set at regular intervals. Metrics like f1, accuracy, precision, and recall are monitored to determine how well the model generalizes to unseen data. If overfitting (high training accuracy but low validation accuracy) or underfitting (poor performance on both training and validation data) is detected, adjustments to the hyperparameters or to the model architecture are made.

#### 3.2.11. Model Testing.

Once training and validation are complete, the model is evaluated on the test set to measure its final performance. This step provides an unbiased assessment of how well the model generalizes to fully unseen data. Metrics like F1-score, confusion matrix, and area under the curve (AUC) are analyzed to gain insights into the model’s overall strengths and weaknesses.

#### 3.2.12. Feedback Loop.

After deployment, user feedback and real-world data are collected and analyzed. This data can be used to:

- Identify areas where the model may require improvement.
- Retrain or fine-tune the model to adapt to changing data distributions or requirements.

The feedback loop ensures the model remains effective and relevant over time, **continually** improving its performance.

## 4. Experiment

### 4.1. Model selection

To evaluate Vietnamese language detection across different architectural paradigms, we selected three distinct Transformer-based models. XLM-RoBERTa is selected for its robust cross-lingual pre-training (trained on 100 languages), which theoretically makes it superior for handling code-switching and mixed-language inputs often found in informal Vietnamese text, BARTPho is Chosen as the primary Vietnamese-specific baseline. Because it is pre-trained on large-scale Vietnamese corpora, it provides a benchmark for capturing language-specific nuances such as complex diacritics and tonal syntax that multilingual models might miss and Multilingual-E5 is Included to assess the viability of dense retrieval models for classification tasks. While primarily optimized for semantic embeddings, its efficiency in encoding semantic meaning offers a potential advantage in processing speed and resource usage compared to generative architectures.

### 4.2. Training preparation

According to the diagram, the Vietnamese category constitutes a major portion of the dataset [22]. We decided to sample 6 million records categorized as Vietnamese and implement a weighted loss function based on the categories to avoid bias. Since our dataset is large enough, we divided it into 2 sets: training set and test set, with the proportion of 80% train and 20% test, where the train set contains 6,701,877 entries and the test set contains 1,675,469 entries.

**Fig 3:**
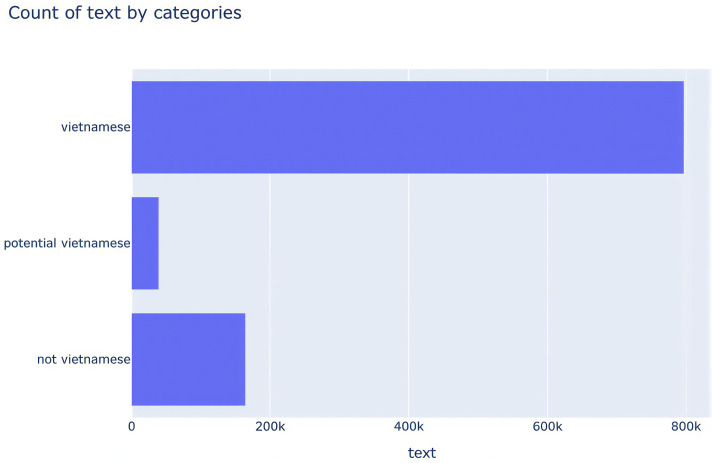
Class Imbalance in the Vietnamese Dataset.

Dataset categories are also encoded using LabelEncoder from the scikit-learn library. To rigorously mitigate the imbalance observed in the class distribution as shown in Fig 3, we calculated specific class weights using the compute_class_weight function with the “balanced” strategy. This method assigns values inversely proportional to the class frequencies, ensuring that minority classes such as “not vietnamese” and “potential vietnamese” contribute more significantly to the gradient updates. These weights were converted to a PyTorch tensor and utilized within a custom WeightedTrainer class, which extends the standard Hugging Face Trainer. By overriding the compute_loss method, we implemented nn.CrossEntropyLoss weighted by the pre-calculated tensor. This approach effectively adjusts the cost function, penalizing the model more heavily for misclassifying underrepresented samples and preventing the optimization process from being dominated by the Vietnamese category.

### 4.3. Training process

#### 4.3.1. XLM-RoBERTa.

We chose XLM-RoBERTa-Base to proceed with the training process, since this model is suitable for simple tasks such as classification. The training process involved 12,500 steps using a batch size of 128 and an initial learning rate of 1e-5. Due to the rapid convergence of the model and the achievement of high accuracy early in the optimization process, we implemented an early stopping strategy. Training was terminated at step 12,500 to prevent overfitting and ensure computational efficiency. The final performance metrics, presented in

**Fig 4:**
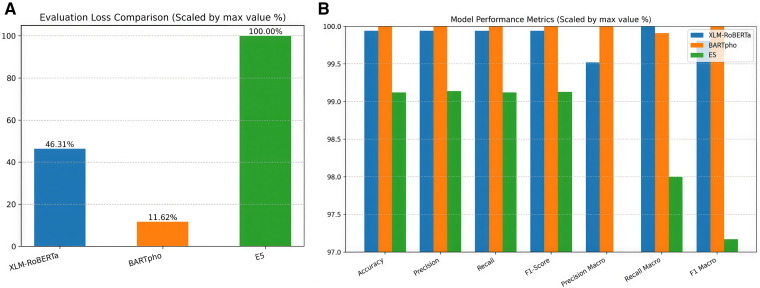
Comparative Model Performance. **(a)** Evaluation Loss scaled relative to the maximum observed loss (E5 = 0.0906, set as 100%). Lower percentages indicate better convergence, with BARTPho achieving only 11.62% of the maximum loss. **(b)** Performance metrics (Accuracy, Precision, Recall, F1) scaled relative to the maximum score achieved across models.

Table 11 and Fig 4, were evaluated on the held-out test set comprising 1,675,469 records.

**Table 11:** Comparison between metric tables of model XLM-RoBERTa, BARTpho, E5.

Metrics	*XLM-RoBERTa*	*BARTpho*	E5
Evaluation Loss	0.041938	0.010520	0.090557
Accuracy	0.996764	0.997360	0.988588
Precision	0.996752	0.997350	0.988797
Recall	0.996764	0.997360	0.988588
F1-Score	0.996757	0.997343	0.988676
Precision Macro	0.991041	0.995823	0.958553
Recall Macro	0.988007	0.987112	0.968261
F1 Macro	0.989515	0.991398	0.963316

#### 4.3.2. BARTPho.

To implement the BARTpho model for text classification, we focused on optimizing its performance for multilingual text processing tasks, including Vietnamese. The training was conducted with careful consideration of computational efficiency and effectiveness. With limited access to hardware, the training process involved 12,500 steps, with a batch size of 128 and an initial learning rate of 1e-5. The evaluation process also follows the same configuration. The training process was concluded at step 12,500. At this checkpoint, the model demonstrated metric convergence, indicating that optimal performance had been achieved. Early stopping was subsequently employed to maximize computational efficiency. The trained model was evaluated on a test dataset consisting of 1,675,469 inputs. The evaluation yielded the key metrics as shown at Table 11 and Fig 4.

#### 4.3.3. E5.

To implement the E5 model for text classification, we selected the Multilingual-E5-Base variant, which is optimized for language processing tasks in Vietnamese. Due to hardware limitations, we conducted the training process with a focus on efficiency and effectiveness. The training process involved 12,500 training steps using a batch size of 128 and an initial learning rate of 1e-5. The dataset was preprocessed to address issues like missing context and mixed language inputs, as observed in prior analyses. The training data consisted of 80% of the total dataset, while the remaining 20% was used for validation. The trained model was evaluated on a separate test set, derived from the earlier split, records. The results demonstrated at Table 11 and Fig 4 show this model metrics.

As illustrated in Fig 4 (Left), the Evaluation Loss metrics reveal significant differences in model convergence that are not immediately apparent in the accuracy scores. To facilitate comparison, the loss values are normalized against the highest observed loss (Multilingual-E5 = 0.0906, set as 100%).

BARTPho demonstrates superior optimization, achieving a relative loss of just 11.62% compared to the baseline. This indicates that BARTPho fits the training data distribution with significantly higher confidence than its counterparts. XLM-RoBERTa follows with a relative loss of 46.31%, while Multilingual-E5 exhibits the highest loss. This disparity highlights that while all models achieve high accuracy (above 98%), BARTPho provides the most probabilistically robust predictions for Vietnamese text.

### 4.4. Model results and metric table comparations

To ensure a rigorous and unbiased comparison between the disparate architectures (XLM-RoBERTa, BARTpho, and E5), we established a unified experimental configuration. A consistent learning rate of 1e-5 was applied across all models. This conservative rate was chosen to facilitate stable fine-tuning, mitigating the risk of catastrophic forgetting—a crucial factor for preserving the rich cross-lingual representations of XLM-RoBERTa and the sensitive semantic embeddings of E5. Regarding the training dynamics, we standardized the global batch size at 128 for all experiments. This substantial batch size ensured accurate gradient estimation and promoted smoother convergence patterns.

Consequently, all models were trained for a uniform training duration of 12,500 steps. This extended training schedule provided sufficient exposure to the dataset, allowing the models to generalize effectively from the training samples while maintaining computational efficiency. This standardized approach ensures that any observed differences in performance can be attributed to the architectural capabilities of the models themselves rather than discrepancies in the training regimen.

The training process of the XLM-RoBERTa model ranks second in training time among the three models, and no issues were encountered during the training and evaluation process. Compared to XLM- RoBERTa and E5, the BARTpho model performs worse than its competitors during evaluation. While evaluating this model, an OOM (out-of-memory) issue occurred even though the configuration was the same as for the other models. The training process of E5 is the fastest, and no performance issues were observed during the training and evaluation process.

## 5. Discussion

### 5.1. Comparative Analysis of Model Architectures

To provide a comprehensive comparative analysis, this section details the specific strengths and limitations of the three implemented models: XLM-RoBERTa, BARTpho, and E5. The evaluation is based on a dual framework of quantitative performance—measured by Accuracy, F1-Score, and Evaluation Loss—and qualitative operational factors. While all three models demonstrated high efficacy in classifying Vietnamese text, they exhibit distinct behaviors when handling specific linguistic nuances and edge cases, as shown in Table 12. These examples illustrate the practical trade-offs between predictive precision and resource efficiency inherent in each architecture.

**Table 12:** Example used for comparing with each model.

Text	Meaning	XLM-RoBERTa	BARTpho	E5	Correct Value
Cô gái đã khiến Phi Nhung lẫn Thanh Hà tranh giành phải oẳn tù tì phân thắng bại	The girl made both Phi Nhung and Thanh Hà compete, having to play rock-paper-scissors to determine the winner.	potential Vietnamese	Vietnamese	Vietnamese	Vietnamese
Cqả Giò ktrái cây	Pork rolls and fruits	potential Vietnamese	potential Vietnamese	Vietnamese	potential Vietnamese
Emerson trái tranh bónf cùng Figo của Inter	Emerson contests the ball with Figo from Inter.	Vietnamese	potential Vietnamese	Vietnamese	potential Vietnamese
Weet je nog	Do you remember?	not Vietnamese	not Vietnamese	Vietnamese	not Vietnamese
Han är astronaut	He is an astronaut.	not Vietnamese	not Vietnamese	Vietnamese	not Vietnamese

#### 5.1.1. XLM-RoBERTa.

- Advantages: Trained on 100 languages, and initially based on RoBERTa, it effectively handles diverse and mixed-language inputs, and its performance across a wide range of NLP tasks is strong enough to deliver reliable results. The pretrained model is widely supported and documented, making it easier to fine-tune. During the training process, this model achieved impressive results with high Accuracy (99.68%) and F1-Score (99.68%), showcasing strong robustness in Vietnamese text classification.
- Disadvantages: This model requires substantial computational resources, but is not as demanding as some other models. While its Evaluation Loss (0.042) is low, it still slightly lags behind the Vietnamese-specific optimizations of BARTpho.

#### 5.1.2. BARTPho.

- Advantages: This model is pretrained on Vietnamese datasets, offering enhanced understanding of syntax and tokenization specific to the language. Built on the BART architecture, this model is versatile for tasks involving sequence-to-sequence generation and classification. During the training process, this model proved to be the superior performer, achieving the highest Accuracy (99.74%) and F1-Score (99.73%). Notably, it achieved the lowest Evaluation Loss (0.0105), indicating the most accurate fit to the dataset.

- Disadvantages: This model is prone to out-of-memory (OOM) issues during evaluation, even on high-end hardware (Nvidia A40) [23]. This leads to increased training time due to the computational complexity of its architecture.

#### 5.1.3. E5.

- Advantages: Optimized for tasks like dense retrieval, this model offers faster training and evaluation compared to other models, and performs well in scenarios requiring the encoding of semantic meanings and embeddings. During the training process, this model achieved respectable Accuracy (98.86%) and F1-Score (98.87%), though it performed slightly worse than the other two candidates.
- Disadvantages: As a general-purpose dense model, it lacks the fine-tuned Vietnamese-specific optimizations of BARTpho. Trailing XLM-RoBERTa and BARTpho with a higher Evaluation Loss (0.0906) and a lower F1 Macro (96.33%), this model is less effective in handling nuanced or complex Vietnamese text classification tasks.

### 5.2. Solutions

As noted, the BARTPho architecture demands substantial computational resources, resulting in Out-Of-Memory (OOM) constraints during the evaluation phase, despite the utilization of high-capacity hardware (NVIDIA A40) [23]. To address this limitation without compromising the integrity of the evaluation or reducing the full test corpus, we implemented a sequential, chunk-based evaluation approach.

Rather than reducing the dataset size, the system was configured to process the test data in discrete segments. Empirical analysis identified an optimal partition size of 20,000 records, establishing a necessary safety buffer below the hardware’s observed saturation point of approximately 26,500 records. Within each segment, memory efficiency was further improved through the use of gradient checkpointing and a batch size of 128.

Furthermore, the pipeline incorporated distinct memory reclamation routines, explicitly invoking garbage collection and clearing CUDA caches between segments to prevent resource accumulation. Finally, to ensure statistical rigor, performance metrics were derived by aggregating raw predictions and ground-truth labels across all segments, rather than averaging segment-level metrics.

Upon inspecting incorrect cases, these occurred because of the lack of context, and there are mixed English or other languages inside the input texts. We can tackle this issue by:

**Further fine-tuning the model** A careful analysis of the incorrect cases shows the model struggles with mixed-language inputs and misspelled Vietnamese. We can improve accuracy by further fine-tuning on datasets specifically containing mixed-language samples and misspellings, enabling the model to better identify Vietnamese when contextual information is limited.

**Preparing our dataset more carefully** Analysis of errors reveals inputs that are ambiguous or contain non-Vietnamese tokens, causing misclassification. We therefore need to clean and curate the data more thoroughly to reduce misleading examples and prevent model confusion.

### 5.3. Expanded error analysis

To understand the models’ decision-making processes beyond aggregate metrics, we conducted a qualitative analysis of misclassified samples. While quantitative explainability methods like SHAP or LIME typically highlight token-level feature importance, our analysis focuses on the linguistic patterns that create ambiguity in the model’s decision boundary.

As shown in Table 12, the primary confusion stems from “orthographic overlap”-where non-diacritical Vietnamese tokens (e.g., “ma”) serve as valid words in other languages (e.g., Spanish or Italian). This suggests that the models rely heavily on unique diacritical markers (e.g., “ư”, “ơ”, “đ”) as primary features for classification. When these markers are absent (non-diacritical text), the models struggle to infer language identity solely from semantic context, leading to lower confidence scores and misclassification in short text segments.

During our analysis of the misclassification instances, we identified specific limitations of the models, primarily stemming from the inherent complexities of the Vietnamese language, especially in informal and non-diacritical contexts. The errors can be categorized as follows:

**Ambiguity in Non-diacritical Text** A significant portion of errors arises when processing Vietnamese text without diacritics. In such cases, a single word can have multiple meanings, making it difficult for the model to distinguish it from words in other languages. For example, the non-diacritical word “ma” could correspond to the Vietnamese words “ma” (ghost), “mợ” (mother), or “mà” (but), or it could be a word in another language. When context is insufficient, the model may misclassify the text.

**Challenges with Code-Switching and Loanwords** In informal communication, particularly on social media, it is common for users to mix Vietnamese and English (code-switching) or use anglicized Vietnamese words. The models sometimes struggle to correctly classify text containing a high density of English loanwords or phrases, occasionally defaulting to a non-Vietnamese classification if the foreign terms dominate the input.

**Informal Language, Slang, and Neologisms** The models are less effective when encountering slang, abbreviations, and newly coined words that are not well-represented in the training data. For instance, “ko” for “không” (no) or “bt” for “biết” (to know) are common in texting and social media. While the models can often infer the language from context, shorter texts with a high density of such informalities pose a significant challenge.

**Confusion with Other Diacritic-Using Languages** Although the models are trained to differentiate Vietnamese from other languages that use diacritics, some confusion can still occur, particularly with short text snippets. Languages such as Spanish or Catalan, which also employ the Latin alphabet with accented characters, can sometimes be misidentified if the specific diacritics present are not uniquely Vietnamese and the surrounding text lacks definitive Vietnamese words.

## 6. Conclusion

This study presents an effective approach for detecting Vietnamese text in both standard and highly challenging informal forms, including non-diacritical, misspelled, and mixed-language inputs. By systematically aggregating multiple large-scale corpora, applying rigorous preprocessing, and performing strategic class balancing, we developed a robust dataset tailored to the complexities of Vietnamese orthography and online linguistic behavior.

Through comparative experiments on three Transformer-based models-XLM-RoBERTa, BARTpho, and E5. Comparative experiments on three Transformer-based models—XLM-RoBERTa, BARTPho, and E5—reveal a critical trade-off between predictive power and computational feasibility. While BARTPho achieved the highest quantitative performance with an Accuracy of 99.74% and F1-Score of 99.73%, it exhibited significant operational instability, causing frequent Out-Of-Memory (OOM) failures even on high-performance hardware (Nvidia A40).

In contrast, XLM-RoBERTa delivered nearly identical accuracy (99.68%) and F1-Score (99.68%) while maintaining complete stability and lower resource overhead. Consequently, we conclude that XLM-RoBERTa represents the most viable state-of-the-art solution for real-world deployment, offering the optimal balance between high-precision detection and the computational efficiency required for scalable applications.

This result highlights the importance of high-quality data preparation and confirms the model’s capacity to capture subtle contextual and orthographic cues even in the absence of tone marks. The dataset and trained models produced in this work establish a strong benchmark and constitute a valuable foundation for advancing Vietnamese language processing in real-world multilingual environments.

Despite these achievements, several limitations remain. Computational constraints restricted the depth of hyperparameter tuning, restricted model training durations, and required reductions in evaluation set size for memory-intensive architectures. Future work will focus on expanding and refining the dataset with more diverse sources, exploring additional modern architectures such as mBERT, mT5, and GPT-based models, and implementing systematic optimization techniques using automated tuning frameworks. We also aim to investigate advanced balancing methods and conduct longer training runs to enable full model convergence.

Addressing these directions will contribute to the development of even more robust and generalizable models for Vietnamese language identification and downstream NLP applications.
